# Treatment strategies for patients with HER2-positive gastric cancer

**DOI:** 10.20892/j.issn.2095-3941.2023.0480

**Published:** 2024-02-05

**Authors:** Feixue Wang, Yi Ba

**Affiliations:** 1 Department of Medical Oncology, Department of Cancer Center, Peking Union Medical College Hospital, Chinese Academy of Medical Sciences, Beijing 100032, China; 2 Department of GI Medical Oncology, Tianjin Medical University Cancer Institute & Hospital, National Clinical Research Center for Cancer, Tianjin’s Clinical Research Center for Cancer, Tianjin Key Laboratory of Digestive Cancer, Key Laboratory of Cancer Prevention and Therapy, Tianjin 300060, China

Gastric cancer (GC) is a major health concern globally, ranking fifth in frequency and fourth in cancer-associated mortality^1^. In China, an estimated 396,500 new cases are diagnosed each year, and late-stage disease comprises more than 80% of cases^2^. Because of late diagnosis and heterogeneous characteristics, the prognosis of GC remains poor. For patients with advanced disease, traditional chemotherapy has been the mainstay of treatment, but its clinical outcomes are far from satisfactory, with a 5-year survival rate below 10%.

In the past several decades, advances in targeted therapy and immunotherapy have achieved unparalleled improvements in precision oncology. For GC, the human epidermal growth factor receptor 2 (HER2, also known as ERBB2) is a major target for drug interventions with promise for future clinical applications. Amplification of ERBB2, can induce overexpression of HER2 protein and lead to the heterodimerization of epidermal growth factor receptor (EGFR) members, activating a series of signaling pathways and promoting tumorigenesis. The success of trastuzumab led to a new era in GC targeted therapy and made HER2-positive subgroup a distinct disease entity, accounting for 15%–20% of all cases of GC. However, following paths after breast carcinoma, the exploration of HER2 targeted therapy in GC is more challenging due to its heterogeneous nature. After many years of effort, therapeutic strategies for HER2-positive GC have greatly evolved.

## Current therapies for HER2-positive GC

### First line treatment: from trastuzumab to immune checkpoint inhibitors

Platinum–fluoropyrimidine chemotherapy has been the gold standard for patients with unresectable and metastatic GC with overall survival less than 1 year. A turning point came in 2010 with the success of the ToGA trial^3^. Trastuzumab, the first humanized monoclonal antibody targeting HER2 receptors, exerts anti-tumor effects *via* inhibiting downstream signaling and antibody-dependent cellular toxicity. In the phase III ToGA trial, addition of trastuzumab to traditional doublet chemotherapy significantly prolonged survival in HER2-positive population (13.8 *vs.* 11.1 months for the overall population; 16.0 *vs.* 11.8 months for the HER2-high subgroup). Since then, combinational trastuzumab with chemotherapy has become a new standard for HER2-positive GC. However, this success was not replicated for the following attempts in HER2 targeted regimens: neither pertuzumab, another humanized monoclonal antibody binding a different epitope on the HER2 receptor, in the JACOB trial^4^, nor lapatinib, a tyrosine kinase inhibitor (TKI) affecting both HER2 and EGFR, in the LOGiC trial^5^, demonstrated clinical efficacy in a first line setting.

Immune checkpoint inhibitors conferred survival promise in advanced GC. Interactions between HER2 and PD-1/PD-L1 identified in preclinical studies led to the hypothesis that combinational immune therapy with an anti-HER2 regimen might exert synergistic anti-tumor effects. The practice-changing study KEYNOTE-811^6^ compared the current standard treatment (trastuzumab plus chemotherapy) with or without pembrolizumab (anti-PD-1) in HER2-positive GC. Interim analysis indicated that the addition of pembrolizumab resulted in a higher objective response rate (ORR, 74.4% *vs.* 51.9%) with an acceptable safety profile. On the basis of this result, pembrolizumab combined with trastuzumab and chemotherapy received U.S. Food and Drug Administration (FDA) approval and has been recommended in clinical guidelines. In a recently released analysis^7^, the median progression free survival (mPFS) and median overall survival (mOS) reached 10.0 months and 20.0 months, respectively, in the pembrolizumab group, compared with 8.1 months and 16.8 months, respectively, in the placebo group. However, no statistically significant difference was found between groups, thus indicating that the FDA-approved indications may need to be narrowed.

Recently developed novel anti-HER2 regimens have heralded new possibilities. Zanidatamab (ZW25) is a bispecific antibody with dual targets of extracellular domain (ECD) II and IV of HER2, the binding sites for trastuzumab and pertuzumab, respectively. In a phase II trial, ZW25 plus tislelizumab (anti-PD1) with chemotherapy was assessed and achieved an ORR of 75% and a disease control rate (DCR) of 100%, which is comparable to the results from KEYNOTE-811. A further head to head phase III trial, HERIZON-GEA-01, comparing ZW25 plus chemotherapy with or without tislelizumab *vs.* the current standard, is ongoing. In the phase II/III MAHOGANY trial^8^, another humanized monoclonal antibody, margetuximab, plus retifanlimab (anti-PD1), has achieved an ORR of 53% in a HER2 3+, PD-L1+ double positive cohort, thus highlighting the possibilities of chemotherapy-free treatment for HER2-positive GC.

### Second line treatment and beyond: antibody-drug conjugates

After the ToGA trial, subsequent investigations on HER2 targets stalled for almost a decade. Given the encouraging performance of trastuzumab as a first line treatment, continued application beyond disease progression gained interest. However, in the T-ACT study^9^, continued application of trastuzumab with paclitaxel did not result in better survival than paclitaxel alone in patients who progressed after first-line trastuzumab with chemotherapy, partly attributing to the HER2 loss identified in a post-exploratory analysis. Recently, a phase I/II study conducted in Korea (HER-RAM trial)^10^ reported different results. More than half of 50 patients after first-line trastuzumab-containing chemotherapy (ORR: 54%) exhibited responses after continuation of trastuzumab plus ramucirumab and paclitaxel. Furthermore, no association between HER2 expression and clinical responses was observed. Thus, whether trastuzumab continuation is applicable requires stronger evidence to be determined. For other anti-HER2 drugs used as second line treatments, lapatinib plus paclitaxel did not exhibit superiority to paclitaxel alone in the TyTAN trial^11^. The novel antibody-drug conjugate (ADC) trastuzumab emtansine (T-DM1), consisting of an anti-HER2 antibody, a cleavable tetrapeptide-based linker, and a cytotoxic topoisomerase I inhibitor, was believed to exert improved efficacy. However, in the GATSBY study, T-DM1 failed to demonstrate superior efficacy over current standard option^12^.

The confirmation of the clinical benefits of trastuzumab deruxtecan (T-DXd), another ADC drug with a bystander effect, in previously treated patients with GC, was a critical second breakthrough in HER2-directed therapy. In the phase II Destiny-Gastric01^13^ study, the investigators compared T-DXd with the physicians’ choice (taxane or irinotecan) as a third line treatment or beyond, and observed a 37% improvement in ORR (51% *vs.* 14%). In the following Destiny-Gastric02^14^ study, conducted in Western populations, T-DXd monotherapy achieved an ORR of 41.8% and a median OS of 12.1 months as a second line treatment, thus accelerating clinical approval of this treatment in Japan, the USA, and Europe. An ongoing head to head investigation in the Destiny-Gastric04 study is exploring treatment efficiency in Asian patients. Moreover, the Chinese domestic ADC drug disitamab vedotin (RC-48) with MMAE linked has demonstrated survival benefits in the second line setting^15^; consequently, RC-48 is currently recommended by the CSCO guidelines for the HER2 2+ population as a third line treatment.

After years of disappointment, HER2-directed therapy in GC treatment achieved a second breakthrough. The novel ADCs T-DXd and RC-48 have revolutionized clinical practice for HER2-positive GC as a second line treatment and beyond. Given the failure of trastuzumab continuation and HER2 expression alterations during treatment, confirmation of HER2 status before the second line treatment is needed for clinical decision-making.

### Moving to perioperative application

The success of HER2-directed therapy in advanced disease promoted further investigations in the perioperative stage. In the NEOHX trial^16^, neoadjuvant therapy with trastuzumab and XELOX improved the R0 resection rate to 78%. Similarly, the R0 resection rate and pCR rate reached 93% and 21.4%, respectively, when trastuzumab was combined with FLOT in the single-arm HER-FLOT study^17^. Unfortunately, both trials failed to transform the improved R0 resection rate and pCR rate into survival benefits. The combination of trastuzumab, pertuzumab, and FLOT also exhibited favorable tumor shrinkage, with a pCR rate of 35%^18^; however, owing to the failure of JACOB trial, it was terminated without final OS result and further chance for phase III trial. To date, no targeted drugs have been approved for perioperative settings in clinical practice. Attempts remain ongoing, and several studies are recruiting participants. Research has not been limited to trastuzumab and pertuzumab; the ADC drug T-DXd is also moving to applications for localized disease. Notably, issues regarding perioperative targeted therapy remain to be determined, including the duration of anti-HER2 medication, before or after surgery, and the “wait and see” approach. There is still a long way to go for perioperative targeted therapy in patients with HER2-positive GC.

## Special considerations in HER2 targeted therapy

### Drug resistance

As described above, continuation of trastuzumab beyond first line treatment has not yet been demonstrated to have clinical efficacy, thus implying the existence of drug resistance. Moreover, 29% to 61% rates of HER2 loss after trastuzumab have been reported in different studies^19^. Medications targeting HER2 might eradicate HER2-expressing tumor cells, thus leading to the dominance of HER2-negative clones. Beyond expression loss, HER2 alterations, genetic and epigenetic factors, are also responsible for the failed inhibition of HER2 signaling. In addition, the activation of alternative pathways (e.g., RAS/MAPK and PI3K/AKT), either present at diagnosis or acquired during treatment, has been found to compensate for the suppressed downstream signaling and to overcome HER2 blockade, thus indicating a possible mechanism for primary and acquired resistance, respectively. Spatial and temporal genomic heterogeneity may explain previous treatment failures.

Given the considerations discussed above, re-evaluation of HER2 expression status is essential for clinical decision-making, particularly for patients progressing after HER2-directed medication. More importantly, with better understanding of the mechanisms underlying drug resistance, strategies have been devised to improve targeted therapy efficiency. On the one hand, combinational strategies are used to enhance the suppression of HER2 signaling. On the other hand, novel anti-HER2 drugs, including antibodies with dual targets, ADCs that exert bystander effects, have been developed to overcome drug resistance.

### Biomarkers for HER2 targeted therapy

Notably, previous explorations of molecular driven treatment for HER2-positive patients encountered obstacles and failures. Only trastuzumab and 2 ADCs achieved successful clinical translation under certain conditions. Tumor heterogeneity and a lack of precise biomarkers are major contributors to previous failures.

The current definition of HER2 positivity is based primarily on the post subgroup analysis of the ToGA trial, in which a much wider survival gap was achieved when the positive population was defined by an immunohistochemical (IHC) score of 3+ or an IHC score of 2+ with positive fluorescence *in situ* hybridization (FISH). Since then, these criteria combined with IHC and FISH based testing methods have been used in clinical practice. Advances in HER2-directed drugs have posed challenges to the present standard. In RC48-C008, recruiting patients with IHC 3+ or 2+ HER2 expression without FISH results, a clinical response was also observed in the HER2 low (IHC 2+) group. Together with the identified correlation between clinical prognosis and the HER2 amplification level, determined through next-generation sequencing (NGS)^19^, an optimized definition of HER2 positivity and detailed patient grouping are necessary for more precise patient selection.

Considering cancer heterogeneity and drug resistance, scientists are now searching for biomarkers for HER2 targeted therapy. For example, co-amplification of receptor tyrosine kinase (RTK) and CCNE1, encoding the cell cycle regulator cyclin E1, has been found to be involved in HER2 drug resistance. In a case-control study, researchers constructed a panel of genomic alterations (AMNESIA panel, including EGFR/MET/KRAS/PTEN mutations and EGFR/MET/KRAS amplifications) and demonstrated that combining HER2 status with the AMNESIA panel improved the accuracy of predicting primary resistance, thus facilitating more precise patient selection. Recently, with developments in NGS and liquid biopsies, novel serum biomarkers have gained interest, because of their clinical convenience. Circulating tumor DNA is a notable candidate bearing alterations consistent with tumor features and clinical parameters. Technological advances have made noninvasive and dynamic monitoring of molecular characteristics along the entire disease course feasible in clinical settings.

## Challenges and future directions

### Escalation or de-escalation

**Figure 1:**
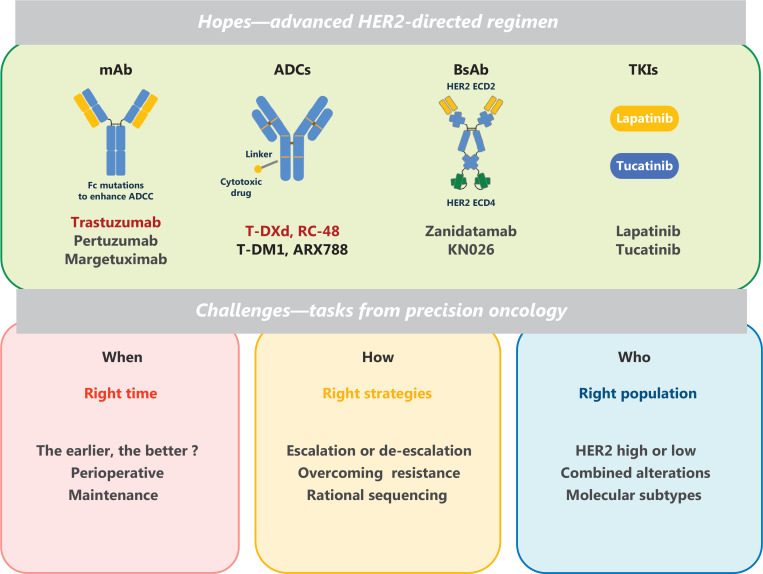
Current developments and challenges in HER2-directed therapy. Drug development targeting HER2 and major tasks in the era of precision medicine. ADC, antibody drug conjugate; BsAb, bi-specific antibody; mAb, monoclonal antibody; TKIs, tyrosine kinase inhibitor.

In the era of precision oncology, determining the right timing and right treatment combination for the right population is the main goal of personalized patient-centric therapy. Decades of efforts have established a broad panel of HER2-directed drugs, including monoclonal antibodies, ADCs, bispecific antibodies, and TKIs (**Table 1**). Performing de-escalation or escalation to optimize the therapeutic combination and layout is a major challenge to be addressed in the future (**Figure 1**).

**Table 1:** Major clinical trials of HER2-directed therapy in gastric cancer

Anti-HER2 regimen		Key trial	Phase	Design	Outcomes	
***Perioperative therapy***						
**mAb**	Trastuzumab	NEOHX^16^	II	Trastuzumab + XELOX	R0 resection rate: 78%; pCR: 8%	2021
		HER-FLOT^17^	II	Trastuzumab + FLOT	R0 resection rate: 93%; pCR: 21.5%	2021
		PETRARCA^18^	II	Trastuzumab + pertuzumab + FLOT FLOT	pCR: 35% *vs.* 12%	2020
		INNOVATION	II	CT Trastuzumab + CT Trastuzumab + pertuzumab + CT	mpRR: 23.3% *vs.* 37.0% *vs.* 26.4% R0 resection rate: 83.9% *vs.* 90.3% *vs.* 85.9%	2019
**ADCs**	T-DXd	EPOC2003	II	T-DXd	Ongoing	NCT05034887
***First line***						
**mAb**	Trastuzumab	ToGA^3^,*	III	Trastuzumab + CT CT	ORR: 47% *vs.* 35%; mOS: 13.8 *vs.* 11.1 m; mPFS 6.7 *vs.* 5.5 m	2010
		KEYNOTE811^6,7^	III	Pembrolizumab + trastuzumab + CT Trastuzumab + CT	ORR: 74.4% *vs.* 51.9%; pCR 11.3% *vs.* 3.1%; mOS: 20.0 *vs.* 16.8 m; mPFS 10.0 *vs.* 8.1 m	2023
	Margetuximab	MAHOGANY^8^	II/III	Margetuximab + retifanlimab	Cohort A (HER2 3+, PD-L1+) ORR: 53%; DCR: 73%	2022
	Pertuzumab	JACOB^4^	III	Pertuzumab + trastuzumab + CT Trastuzumab + CT	ORR: 56.7% *vs.* 48.3%; mOS: 17.5 *vs.* 14.2 m; mPFS 8.5 *vs.* 7.0 m	2018
**BsAb**	Zanidatamab (ZW25)	BGB-A317-ZW25-101	Ib/II	ZW25 + tislelizumab + CT	ORR 75.8%, DCR 100% mPFS 10.9 m	NCT04276493
		HERIZON-GEA-01	III	ZW25 + CT *±* tislelizumab Trastuzumab + CT	Ongoing	2022
	KN026	KN026-203^20^	II	KN026 + KN046	ORR: 77.8%; DCR: 92.6%	2023
**TKIs**	Lapatinib	LOGiC^5^	III	Lapatinib + XELOX XELOX	ORR: 53% *vs.* 39%; mOS: 12.2 *vs.* 10.5 m; mPFS: 6.0 *vs.* 5.4 m	2016
***Second line***						
**mAb**	Trastuzumab	T-ACT^9^	II	Paclitaxel + trastuzumab Paclitaxel	ORR: 31.6% *vs.* 33.3%; mOS: 10.0 *vs.* 10.2 m; mPFS: 3.2 *vs.* 3.7 m	2020
		HER-RAM^10^	Ib/II	Trastuzumab + ramucirumab + paclitaxel	ORR: 54%; DCR: 96%; mOS: 13.6 m; mPFS: 7.1 m	2023
**ADCs**	T-DXd	Destiny-Gastric02^14,^*	II	T-DXd	ORR: 41.8%; mOS: 12.1 m; mPFS: 5.6 m	2023
		Destiny-Gastric04	III	T-DXd Taxane + ramucirumab	ongoing	NCT04704934
	RC-48	RC48-C008^15^,*	II	RC-48	ORR: 24.8%; mOS: 7.9 m; mPFS: 4.1 m	2021
	T-DM1	GATSBY^12^	II/III	T-DM1 Paclitaxel/docetaxel	ORR: 20.6% *vs.* 19.6%; mOS: 7.9 *vs.* 8.6 m; mPFS: 2.7 *vs.* 2.9 m	2017
**BsAb**	KN026	KN026-202	II	KN026	Cohort 1 (HER2 +): ORR: 56%; mPFS: 8.3 m; mOS: 16.3 m	NCT03925974
**TKIs**	Lapatinib	TyTAN^11^	III	Lapatinib + paclitaxel Paclitaxel	ORR: 27% *vs.* 9%; mOS: 11.0 *vs.* 8.9 m; mPFS: 5.4 *vs.* 4.4 m	2014
	Tucatinib	MOUNTAINEER02	II/III	Trastuzumab + tucatinib + ramucirumab + CT	Ongoing	NCT04499924
***Third line and beyond***						
**mAb**	Margetuximab	CP-MGAH22-05^21^	Ib/II	Margetuximab + pembrolizumab	HER2 3+, PD-1+ cohort; ORR: 44%	2020
**ADCs**	T-DXd	Destiny-Gastric01^13^	II	T-DXd Paclitaxel/irinotecan	ORR: 51% *vs.* 14%; mOS: 12.5 *vs.* 8.4 m; mPFS: 5.6 *vs.* 3.5 m	2020

ADC, antibody drug conjugate; BsAb, bi-specific antibody; CT, chemotherapy; DCR, disease control rate; FLOT, 5-fluorouracil, leucovorin, oxaliplatin, docetaxel; m, months; mAb, monoclonal antibody; mPFS, median progression free survival; mOS, median overall survival; mpRR, major pathological response rate; ORR, objective response rate; pCR, pathological complete response; TKIs, tyrosine kinase inhibitor; XELOX, oxaliplatin, capecitabine. *Associated treatment recommended by clinical guidelines.

To date, multiple treatment combinations are under exploration. For example, tucatinib, a highly selective HER2-directed TKI with superior anti-tumor activity in preclinical studies, has been evaluated in combination with trastuzumab, ramucirumab, and paclitaxel in previously treated HER2-positive gastric or gastroesophageal junction cancer. The bispecific antibody zanidatamab (ZW25) has exhibited stronger *in vitro* and *in vivo* anti-tumor activity than trastuzumab at different HER2 expression levels. Following the pattern of current standard, ZW25 combined with chemotherapy has demonstrated improved clinical responses, with an ORR of 75% as a first line treatment for gastroesophageal adenocarcinoma and has been granted orphan drug for GC by the FDA.

Among treatment combination patterns, chemotherapy-free modalities are favorable because of their safety. Margetuximab plus pembrolizumab has achieved a 44% ORR with a manageable safety profile in previously trastuzumab-treated gastroesophageal adenocarcinoma with dual positivity of HER2 (IHC 3+) and PD-L1 (CPS ≥ 1 by IHC)^21^. These encouraging results spurred the MAHOGANY trial comparing margetuximab plus retifanlimab with or without chemotherapy as a first line treatment. An ORR of 53% and DCR of 73% were reported in the chemotherapy-free arm. In addition, another phase II trial, KN026-203, has evaluated the safety and efficacy of combined treatment with the bispecific antibodies KN026 and KN046, and indicated a 77.8% ORR and 92.6% DCR in a GC cohort in the preliminary analysis^20^. Together, these results have shed light on chemotherapy-free treatment of HER2-positive GC.

### Translational research breaking barriers

In parallel with clinical studies, basic and translational investigations including genomics, proteomics, transcriptomics, and immunogenomics are equally important for developing precise oncology^22^. Understanding of the extensive molecular landscape can provide directions and rationale for clinical practice, including patient selection, combination strategies, and overcoming treatment resistance. Technological advances, such as NGS, big data, and bioinformatic algorithms, have made substantial contributions to in-depth understanding of the molecular landscapes of HER2-positive GC. For example, in the JACOB trial, although no significant OS benefit was identified, post hoc translational analysis indicated that HER2-high CNV, assessed by NGS, was associated with better clinical outcomes; therefore, more precise population selection will be necessary in future studies. In addition, by performing whole exome sequencing on paired tumor tissue before and after trastuzumab, Xu et al.^23^ have highlighted the positive correlation between higher chromosome instability and prolonged survival. Given that tumor heterogeneity is a major barrier to precision medicine in GC, more extensive, deeper understanding of the molecular profile of HER2-positive GC would greatly facilitate clinical practice.

On the basis of previous experiences, including both successes and failures, more precise trial design with novelty and reasonable rationales will be highly important. Selecting appropriate populations, timing, and combinational patterns remain major challenges in precision medicine. Integrating extensive translational and preclinical exploration with multi-omics-based clinical trials will be key to future development.

## Conclusions

After decades of pitfalls and promises, HER2-directed therapy has revolutionized the treatment landscape for HER2-positive GC. Newly developed drugs provide new possibilities and promises, yet also pose new clinical challenges, such as drug resistance, patient selection, and therapeutic sequencing. Optimizing clinical algorithms to maximize treatment efficacy and precision remains a major challenge to researchers in the era of personalized medicine and a major priority in the upcoming decades.
